# *LAMA2* variants associated with muscular dystrophy, brain structural abnormalities, and epilepsy: a genotype-phenotype study

**DOI:** 10.3389/fneur.2025.1728652

**Published:** 2026-01-06

**Authors:** Jian Zha, Ying Yu, Fangfang Cao, Zhaoshi Yi, Huaping Wu, Yong Chen, Jianmin Zhong, Xiongying Yu

**Affiliations:** 1 Department of Neurology, Jiangxi Provincial Children’s Hospital, Nanchang, Jiangxi, China; 2 Department of Neonatology, Jiangxi Provincial Children’s Hospital, Nanchang, Jiangxi, China

**Keywords:** brain structural abnormalities, congenital muscular dystrophy, epilepsy, *LAMA2*, neurogenetics

## Abstract

**Background:**

*LAMA2*-related congenital muscular dystrophy (*LAMA2*-MD) is a genetically heterogeneous disorder defined by progressive muscle weakness, brain structural abnormalities, epilepsy, and multisystem involvement. The primary goal of this study was to characterize the clinical features, temporal progression, and genotype-phenotype correlations of *LAMA2*-MD.

**Methods:**

Medical records of patients with genetically confirmed *LAMA2*-MD were extracted from a clinical data repository and analyzed retrospectively. Clinical manifestations, laboratory findings, and neuroimaging features were systematically reviewed and compared across different age groups. Variant data were retrieved from public databases to perform comprehensive genetic analyses.

**Results:**

A total of five patients (two males and three females) were enrolled, delayed motor milestones and varying degrees of ankle contractures and persistent motor impairment in all patients were the initial presenting symptom at diagnosis in all cases, and two patients also exhibited cognitive delays. Laboratory analysis of muscle enzymes showed varying degrees of abnormalities, with creatine kinase (CK) levels displaying the most significant elevation. Cranial magnetic resonance imaging (MRI) revealed symmetrical white matter abnormalities in four patients. Seizures were documented in three school-aged patients. All patients carried compound heterozygous variants in the *LAMA2* gene. A literature review indicated that the most common variant types were stop-gain and missense variants: stop-gain variants were predominantly associated with complete merosin deficiency (MDC1A), whereas missense variants typically correlated with late-onset limb-girdle muscular dystrophy.

**Conclusion:**

*LAMA2*-MD exhibits a broad phenotypic spectrum and a progressive disease course. Early manifestations include muscle weakness, delayed achievement of developmental milestones, joint contractures, seizures and characteristic intracranial abnormalities.

## 1 Introduction

Congenital muscular dystrophy type 1A (MDC1A) is an autosomal recessive disorder caused by variants in the laminin alpha 2 chain gene (*LAMA2*). As the most common subtype of congenital muscular dystrophy (CMD), it accounts for 30% to 50% of all CMD cases (1–3). A study conducted in China further reported that approximately 36.4% of CMD patients are diagnosed with MDC1A (4). The core clinical manifestations of MDC1A include muscle weakness, elevated serum creatine phosphokinase (CPK) levels, inability to ambulate independently, and cerebral white matter abnormalities. These symptoms can progress to respiratory insufficiency which was a major contributor to early mortality in affected patients.

MDC1A exhibits high clinical heterogeneity in severity which presents as neonatal-onset merosin-deficient muscular dystrophy (the classic MDC1A phenotype) or a milder form, such as childhood- or adult-onset autosomal recessive limb-girdle muscular dystrophy 23 (LGMDR23) (5). To encompass this phenotypic spectrum, some researchers have proposed the umbrella term “*LAMA2*-related muscular dystrophy (*LAMA2*-MD)” (6). Seizures have been documented in 8% to 20% of *LAMA2*-MD patients, and cerebral white matter abnormalities typically involve periventricular and subcortical regions.

Epidemiologically, the prevalence of the LGMDR23 phenotype is estimated to range from 1 in 16,000 to 1 in 21,500 individuals, while the incidence of CMD caused by complete or partial merosin deficiency is approximately 0.7 to 2.5 per 100,000 people. However, the exact incidence of *LAMA2*-MD as a whole remains unestablished, and its clinical phenotypic variability and temporal progression continue to be active areas of clinical investigation.

In this study, we retrospective analyzed the clinical data, diagnostic and therapeutic processes of *LAMA2*-MD patients in order to characterize the disease’s clinical heterogeneity and temporal evolution.

## 2 Methods

### 2.1 Study population

This retrospective study included patients with genetically confirmed *LAMA2*-related congenital muscular dystrophy (*LAMA2*-MD) who were evaluated at Jiangxi Provincial Children’s Hospital (Nanchang, Jiangxi, China) between January 2010 and June 2024. Clinical data were collected from both outpatient and inpatient medical records.

### 2.2 Clinical data collection

Detailed clinical data were extracted from medical records including: presenting symptoms and medical history; findings from physical examinations and laboratory tests for muscle enzymology [alanine aminotransferase (ALT), aspartate aminotransferase (AST), lactate dehydrogenase (LDH), creatine kinase (CK), and CK-MB]; cranial magnetic resonance imaging (MRI) results; and cardiac ultrasound findings.

### 2.3 Literature and database review

A systematic literature search was conducted to compare our findings with previously reported cases. Relevant studies published between January 2010 and June 2024 were retrieved from both domestic (Chinese) and international databases.

For Chinese-language literature, searches were performed in the China National Knowledge Infrastructure (CNKI), Wanfang Data, and VIP Chinese Journal Database using the keywords: “congenital muscular dystrophy type 1A” “*LAMA2* gene” and “laminin-α2.” For international literature, PubMed was searched with the keywords: “congenital muscular dystrophy type 1A (CMD1A),” “*LAMA2* gene,” and “merosin.”

To systematically summarize genetic variants of *LAMA2*, the ClinVar, DECIPHER, and Ensembl databases were queried using the search term “*LAMA2*.” Reported pathogenic and likely pathogenic variants, along with their associated phenotypic characteristics, were extracted and analyzed.

### 2.4 Ethics statement

This study was approved by the Medical Ethics Committee of Jiangxi Children’s Hospital (Approval number: JXSETYY-YXKY-20240189). Written informed consent was obtained from the parents or legal guardians of all pediatric patients.

### 2.5 Statistical analysis

Data analysis was performed using SPSS version 26.0 software. Descriptive analysis was conducted for each observed variable. Numerical variables were expressed as mean ± standard deviation (SD), along with minimum and maximum values.

## 3 Results

### 3.1 Patient characteristics

Five patients (two males and three females) were genetically diagnosed with *LAMA2*-MD, the age at first diagnosis ranged from 3 months to 78 months (Median = 37.47, IQR = 4.29–58.32, Range = 3.10–76.63). Four of them were first evaluated in neurology department, and one in rehabilitation department. Patient 1 was first diagnosed in the rehabilitation department due to motor development delay. Three years later, she was hospitalized in the department of neurology. Patient 2 was admitted to the hospital for treatment at 3 years and 4 months due to recurrent seizures for 5 months. Over the next 9 years, the patient was hospitalized more than 20 times due to recurrent epileptic symptoms. The other three patients did not have long-term and detailed records due to cooperation of their families.

### 3.2 Clinical phenotypes

All five patients exhibited motor developmental delay, with motor milestones of different degrees were delayed. Three patients experienced more than two seizures before this study. Their seizures were characterized by sudden loss of consciousness, followed by bilateral tonic–clonic activity lasting 1–3 min. All patients did not capture the ictal EEG, interictal EEG with a slow background, but there was no typical epileptic discharge. In addition, neurological physical examination revealed varying degrees of ankle contractures and persistent motor impairment in all patients.

### 3.3 Muscle enzyme analysis

**Figure 1:**
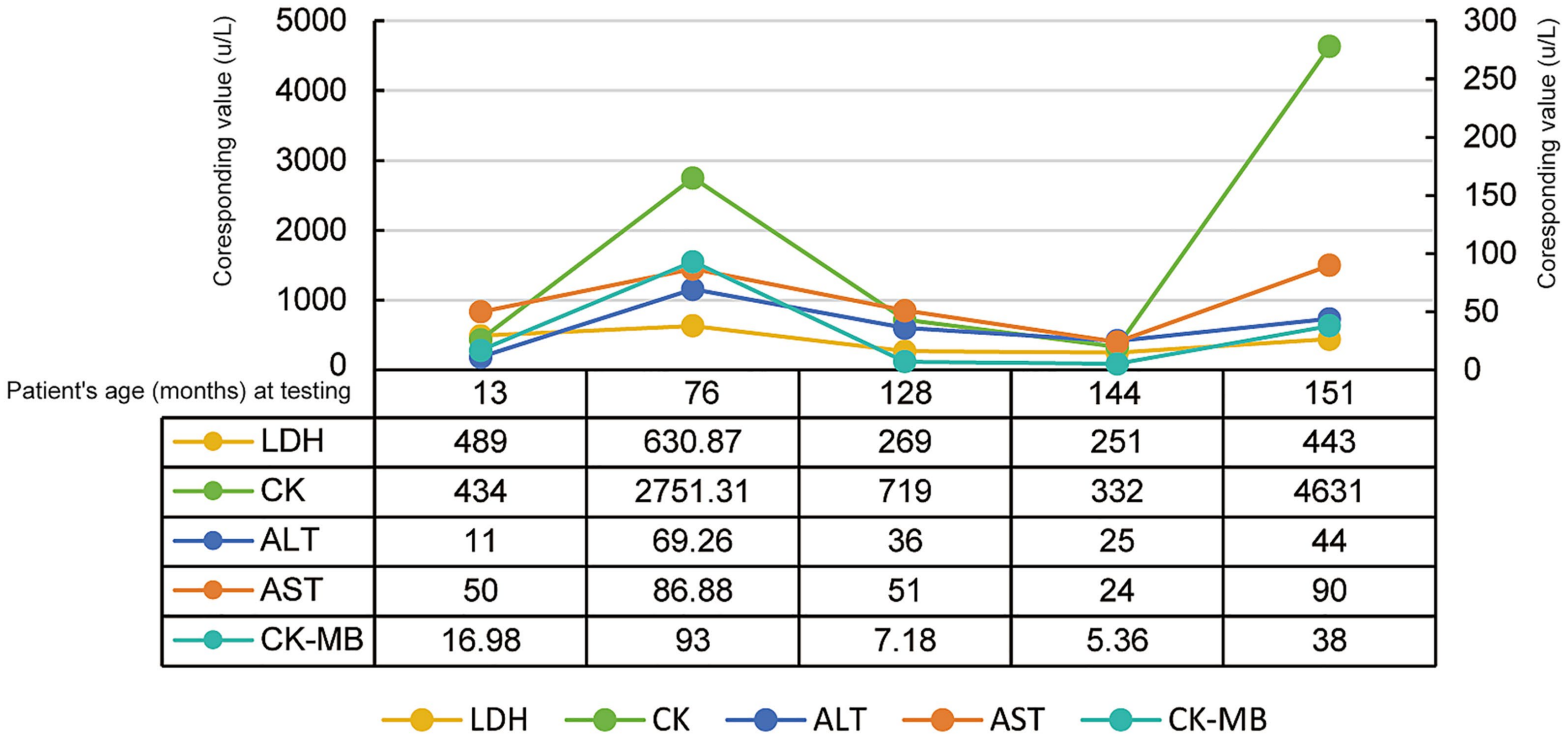
Muscle enzymatic test results in all cases (sorted by test time). CK and LDH correspond to the primary ordinate, CK-MB, AST, and ALT correspond to the secondary ordinate.

All five patients demonstrated elevated muscle enzyme (Table 1). Among them, CK exhibited most significant affected with levels ranged from 332u/L to 4631u/L (Median = 719.00, IQR = 403.00–3691.16, Range = 332.00–4631.00) (Figure 1).

**Table 1:** Clinical data of all the enrolled cases.

No		Case 1	Case 2	Case 3	Case 4	Case 5
ID		8600303632	10698019	100987250	8601381726	8601550748
Gender		F	F	M	M	F
Age of first diagnostic		37.47	5.47	40.00	3.10	76.63
First presentation symptoms		Motor development is 4 years behind	Overall development is 6 years behind	Motor development lags behind the muscle enzyme abnormalities for 3 years	Motor development is 3 months behind	Walking is difficult for 5 years
Seizure		+	+	+	−	−
Muscle Enzyme	ALT	36	25	44	11	69.26
	AST	51	24	90	50	86.88
	LDH	269	251	443	489	630.87
	CK	719	332	4,631	434	2751.31
	CK-MB	7.18	5.36	38	16.98	93
	Detection time	128 months	144 months	151 months	13 months	76 months
Cranial MRI	Result	+	+	+	−	+
	Detection time	23-08-2019	15-03-2023	17-12-2020	11-08-2023	08-06-2023
Variant type		c.1027+3A>G maternal; c.1147delC, p.Gln383Lysfs*6 paternity	c.3955C>T, p.Arg1319* maternal; c.5476C>T, p.Arg1826* paternity	c.7899-1G>A maternal; c.7810C>T, p.Arg2604* paternity	c.5095del, p.Leu1699* maternal; c.7147C>T, p.Arg2383* paternity	c.2272C>T p.Glu758* maternal; c.6624G>C, p.Trp2208Cys; paternity
Developmental milestone		The patient achieved independent walking at 2 years of age, presenting with an unsteady gait. At 10 years and 8 months old, the patient remained ambulatory but was prone to falls, with weak gait motor function, difficulty in squatting and standing up, and exertional climbing of stairs.	Independent walking was attained at 3 years of age, accompanied by gait instability. The patient exhibited progressive motor regression and lost the ability to walk independently at approximately 10 years old.	The patient started walking at around 18 months of age without significant motor regression. Currently, the patient remains ambulatory, with motor function slightly delayed compared to age-matched peers.	The patient was admitted at 1 year of age due to young age-related developmental concerns. At admission, the patient had unstable head control and was unable to sit independently or walk with support.	Independent walking was achieved at approximately 2 years of age, with an unsteady gait and no obvious motor regression. Currently, the patient experiences exertional climbing of stairs and is prone to falls.
Diagnosis and treatment process		Due to backward development (rehabilitation Department in January 2015), I visited the Rehabilitation Department (4 times), abnormal muscle enzymes, skull MRI found diffuse leukoplakia, adrenal leukodystrophy; 7 years and 7 months from 1 month (March 2019) to the neurology department for LAMA 2-MD	Due to backward motor development (first neurology department in September 2017) (12 visits), LAMA 2-MD was diagnosed at Fudan University in October 2017, and the first seizure in December 2020, GTCS and head MRI were mostly diagnosed by metabolic encephalopathy and mitochondrial encephalomyopathy	Due to backward motor development (September 2011), she was diagnosed in the neurology clinic (20 visits) for central coordination disorder and cerebral paralysis. In December 2020, she was admitted to the Neurology Department for repeated convulsions for half a year, with abnormal head MR and abnormal muscle enzyme, and was diagnosed as epilepsy and limb band muscular dystrophy	Due to backward motor development, the first neurology clinic in October 2022 was “congenital muscular dystrophy” (12 visits)	Due to backward motor development and walking difficulties, the first diagnosis was in the neurology clinic in May 2023, and “congenital muscular dystrophy” (7 visits) was genetically diagnosed as LAMA 2-MD, and given hormone therapy, and the symptoms improved

Head MRI “+” indicates: bilateral symmetrical generalized white matter abnormalities; the first three categories were recorded in medical records, and the specific genetic variation type and reports cannot be collected.

### 3.4 Neuroimaging findings

**Figure 2:**
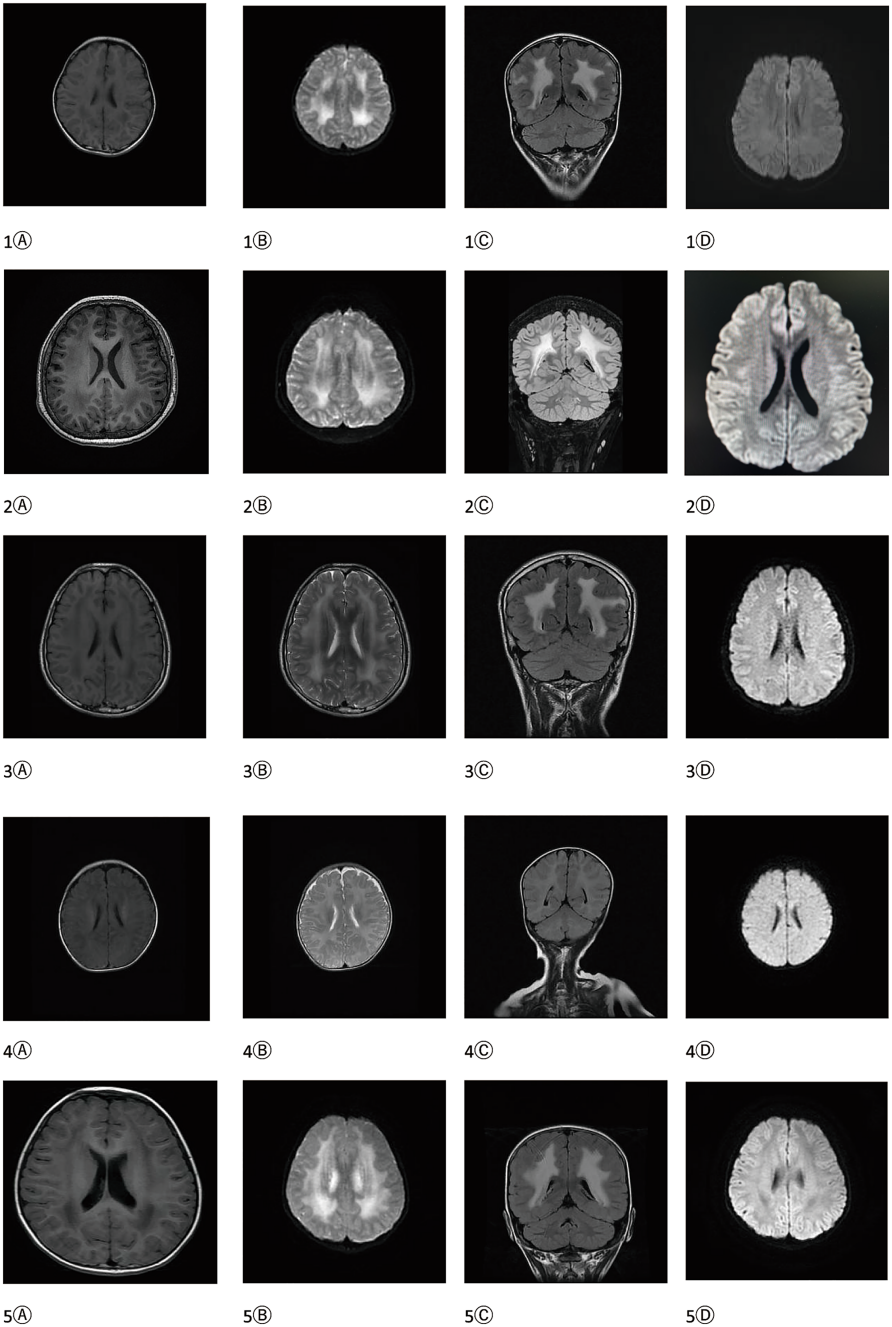
Cranial MRI of all the enrolled patients. Roman numerals indicate the case number, Ⓐ T1W sequence, Ⓑ T2W sequence, Ⓒ T2Flair sequence and Ⓓ DWI sequence.

All enrolled patients underwent cranial MRI, including T1W, T2W, T2-Flair, DWI, and ADC sequences, with crown, transverse, sagittal views. Four patients exhibited bilateral symmetrical white matter signal abnormalities, most pronounced on T2W, and T2-Flair sequences, while DWI showed no obvious diffusion limited performance (Figure 2). Due to the inconsistent time of MRI in five patients, the age of the child with no white matter lesions was detected in the one patient (1 year and 1 month) (Table 1).

### 3.5 Variants evaluation

**Figure 3:**
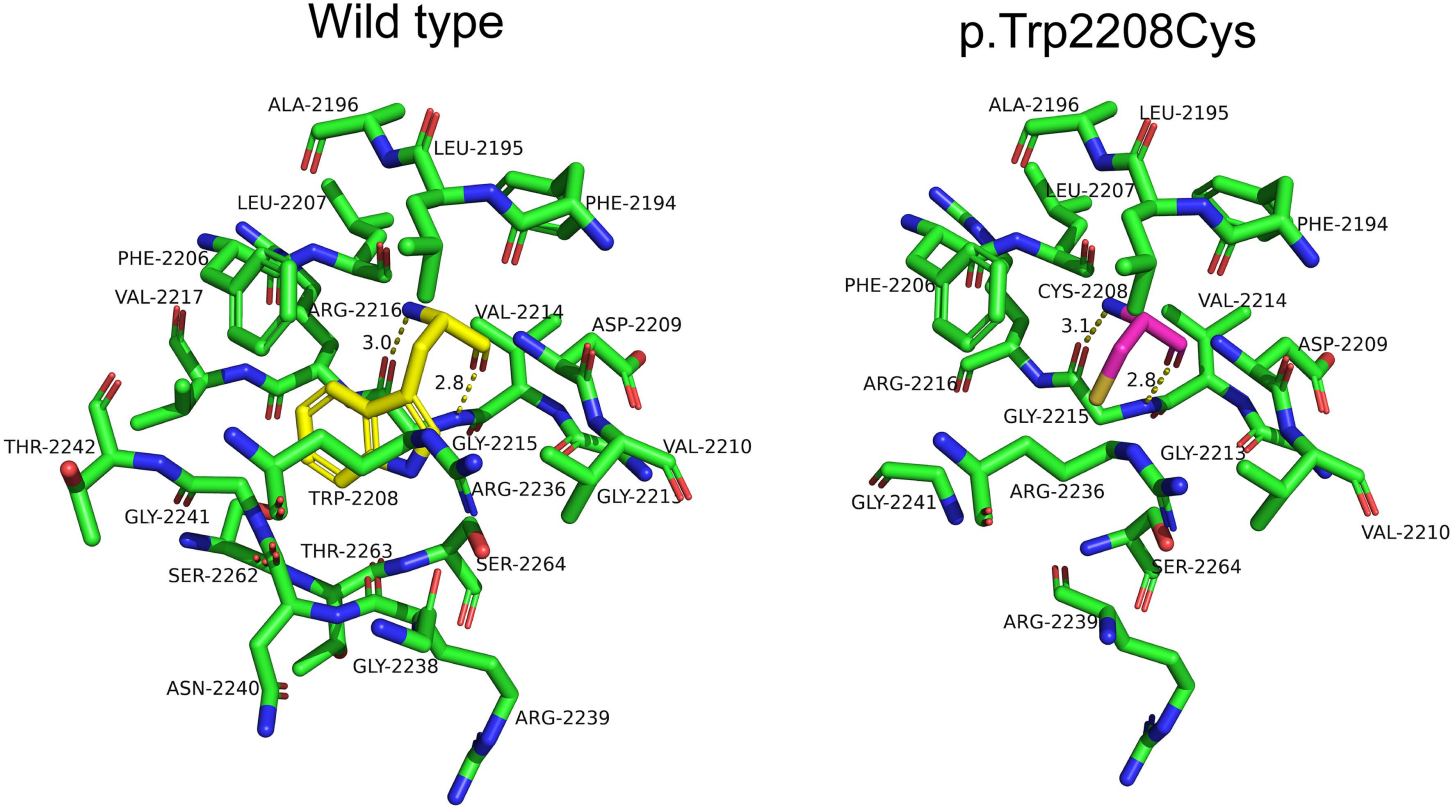
LAMA2 protein 3D structure of wild-type and mutation in p.Trp2208Cys. The residue 2,208 was labeled in yellow in wildtype (left) and purple in mutation (right). The hydrogen bond was labeled with yellow dotted box.

The variants for Case 1 were a splicing variant [c.1027+3 A>G, which predicted to influence acceptor loss (SpliceAI score:0.6) and donor loss (SpliceAI score:0.71)] and a frameshift deletion variant (c.1147delC, p.Glu383Lysfs*6, which predicted to a substitute from Glu to Lys at 383th and generate an early stop codon six amino acids later of *LAMA2*). The variants for Case 2 were two nonsense variants at 1,319 and 1,826, respectively, (c.3955C>T, p.Arg1319*, c.5476C>T, p.Arg1826*). Variants for case 3 were a splicing variant [c.7899-1 G>A, which predicted to influence acceptor loss (SpliceAI score:0.99) and acceptor gain (SpliceAI score:0.89)] and a nonsense variant at 2604 (c.7810C>T, p.Arg2604*). The variants for Case 4 were also two nonsense variants which located at 1,699 and 2,383, respectively, (c.5095del, p.Leu1699*, c.7147C>T, p.Arg2383*). Variants for case 5 were a nonsense variant at 758th (c.2272C>T, p.Glu758*) and a missense variant (c.6624G>C, p.Trp2208Cys) which was collected in ClinVar database as Uncertain significance (VUS). Further analysis revealed the tryptophan to cysteine substitution at position 2,208 led to an increase in the hydrogen bond between residue 2,208 and 2,215 from 3.0Å to 3.1Å, which may affect the stability and function of the protein (Table 1; Figure 3). Among all variants, one variants from patient 1 (c.1147delC, p.Glu383Lysfs*6), one from patient 4 (c.5095del, p.Leu1699*) and one variant from patient 5 (c.2272C>T p.Glu758*) were not collected in ClinVar database or reported in published paper. No other CNVs were observed based on the whole exon sequencing data, analysis method was described as previous study (7). The detailed information such as variant type, genome location, ACMG classification were also listed in Tables 1, 2.

**Table 2:** Genetic data of all the enrolled cases.

Patient	Chromosome	Genome location	Reference	Alteration	Gene	Nucleotide change	Amino acid change	Origin	ACMG evidence	ACMG classification
Patient 1	chr6	129149099	A	G	LAMA2	c.1027+3A>G		Maternal	PM3_Strong+PM2_Supporting+PP3	LP
Patient 1	chr6	129154624	C	—	LAMA2	c.1147delC	p.Gln383Lysfs*6	Paternity	PVS1 + PM2_Supporting	P
Patient 2	chr6	129316068	C	T	LAMA2	c.3955C>T	p.Arg1319*	Maternal	PVS1 + PM2_supporting+PM3_Strong	P
Patient 2	chr6	129401254	C	T	LAMA2	c.5476C>T	p.Arg1826*	Paternity	PVS1 + PM2_Supporting+PM3_VeryStrong+PP1	P
Patient 3	chr6	129491900	G	A	LAMA2	c.7899-1G>A		Maternal	PVS1 + PM3 + PM2_Supporting	P
Patient 3	chr6	129486534	C	T	LAMA2	c.7810C>T	p.Arg2604*	Paternity	PVS1 + PM3 + PM2_Supporting	P
Patient 4	chr6	129391514	AC	A	LAMA2	c.5095del	p.Leu1699*	Maternal	PVS1 + PM2_Supporting+PM3	P
Patient 4	chr6	129464444	C	T	LAMA2	c.7147C>T	p.Arg2383*	Paternity	PVS1 + PM2_Supporting+PM3_VeryStrong+PP1	P
Patient 5	chr6	129267169	C	T	LAMA2	c.2272C>T	p.Glu758*	Maternal	PVS1 + PM2_Supporting	LP
Patient 5	chr6	129454205	G	C	LAMA2	c.6624G>C	p.Trp2208Cys	Paternity	PM2_Supporting+PP3_Moderate+PM3	VUS

### 3.6 Literature review and genotype–phenotype correlation

**Figure 4:**
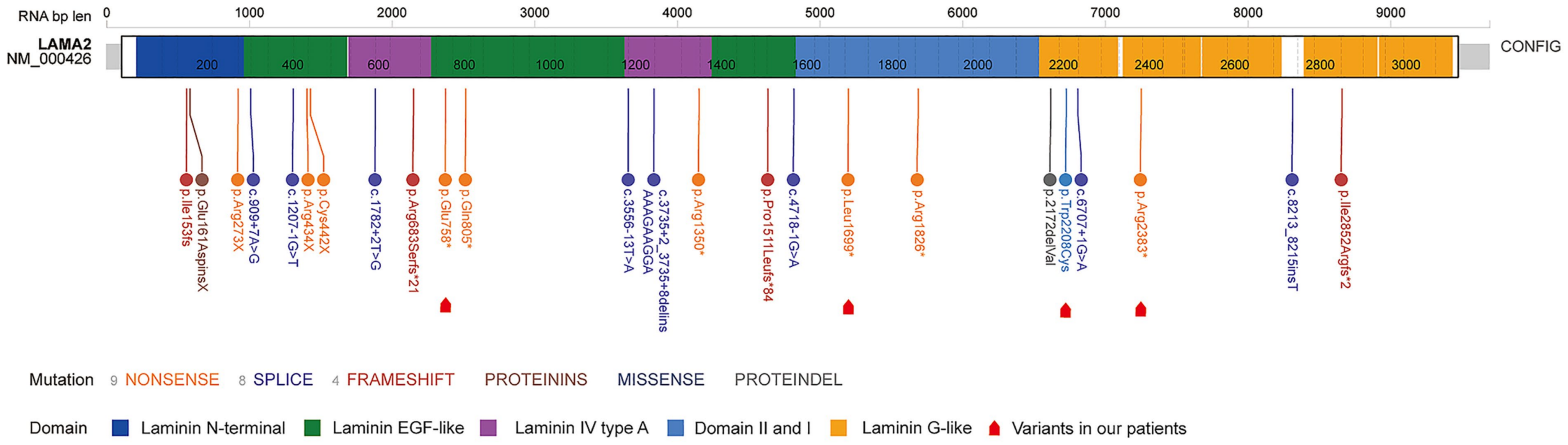
Schematic representation of the gene variants in the enrolled patients. The variants in our patients were highlighted through red arrows.

**Figure 5:**
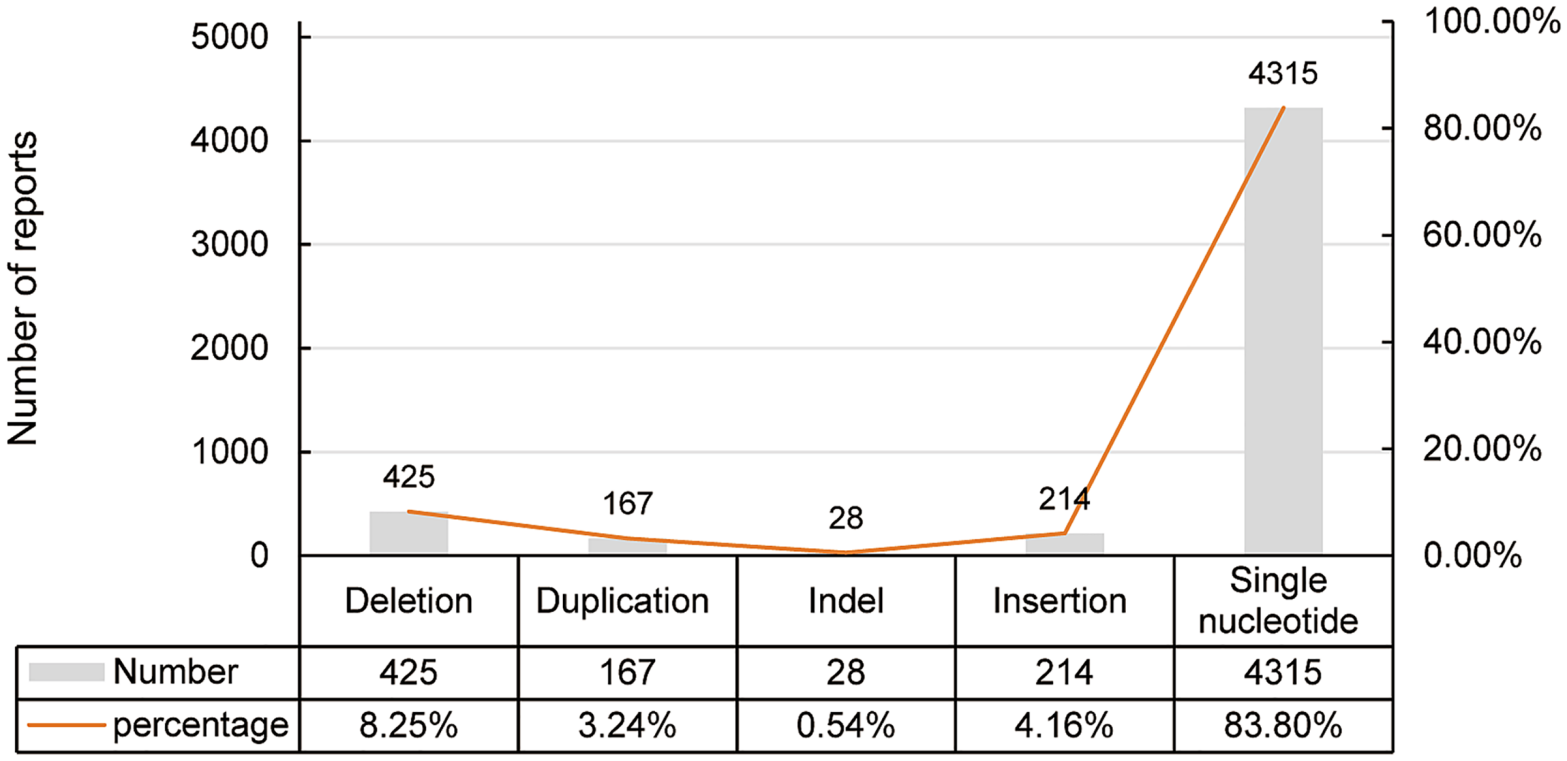
The LAMA2 variant types are reported in the ClinVar database.

**Figure 6:**
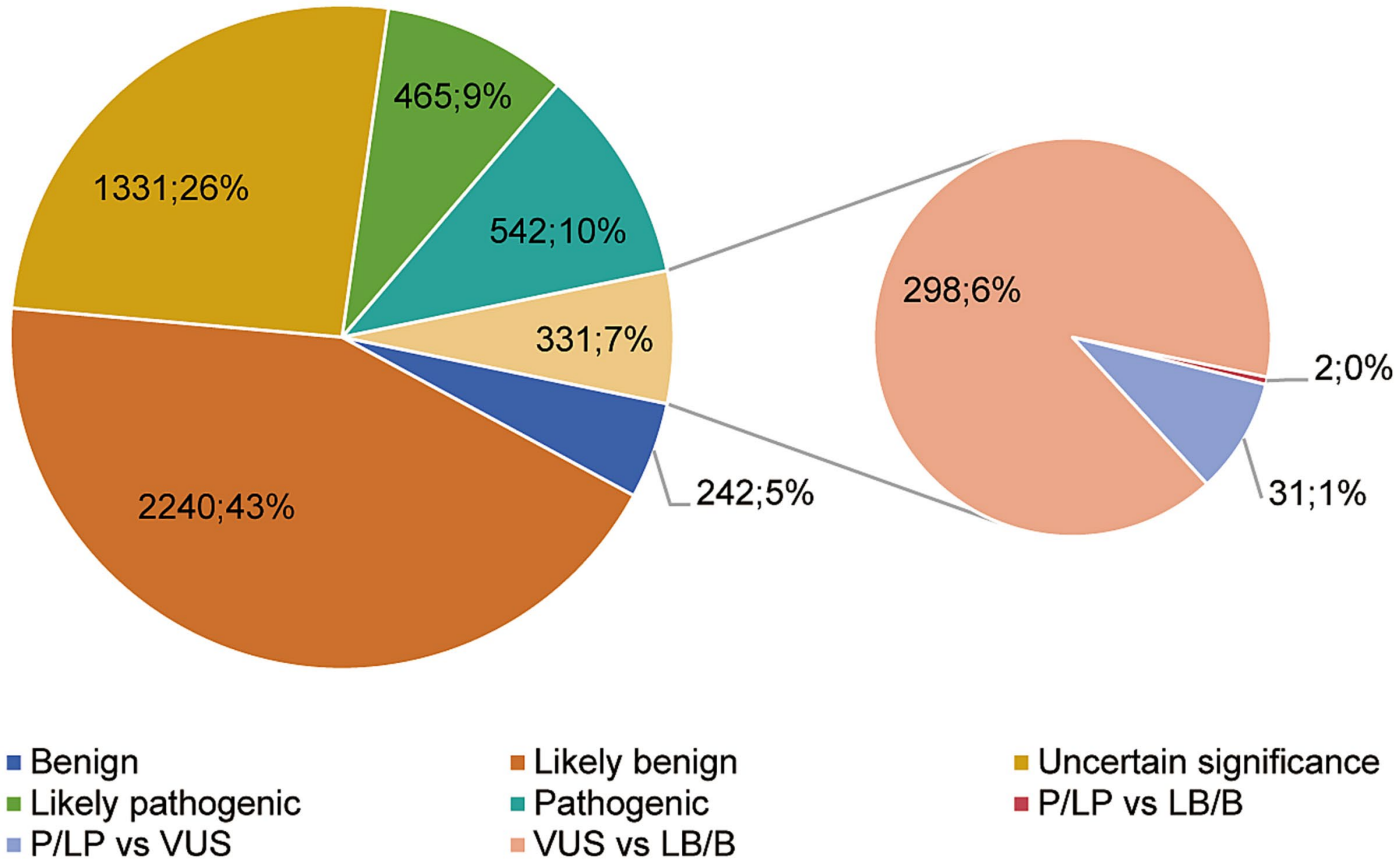
Interpretation of LAMA2 molecular variation results in the ClinVar database.

All enrolled patients harbored compound heterozygous variants in *LAMA2*, the variant sites and affected domains were visualized (Table 1; Figure 4). Up to May 27, 2024, the ClinVar database collected 5,149 variants including 425 deletion, 167 duplication, 28 InDel, 214 insertion and 4,315 single nucleotide variants (Figure 5). Among them, only 542 (10.5%) and 465 were classified as pathogenic (P) or likely pathogenic (LP) variants (9.0%) respectively (Figure 6, data from statistic of ClinVar database). There were 28 reports in the Decipher database, including 20 copy number variants, five SNVs variants, one triploids case, and two single diploids cases. Expression data from the Human Protein Atlas (HPA) showed that *LAMA2* was widely expressed in tissues and organs. The GTEx database showed the highest expression time in the fetal stage, followed by adulthood, and in the blastocyst stage.

In our patients, all of them carried the variants that affect protein structure (stop-gain, frame-shift, or splicing variant) and exhibited early-onset muscular dystrophy and core motor disorders, accompanied by white matter abnormalities (4/5 patients). After review related literatures, we found that the most common variant types were stop-gain and missense variants: stop-gain variants were predominantly associated with complete merosin deficiency (MDC1A), whereas missense variants typically correlated with late-onset limb-girdle muscular dystrophy. This was basically consistent with the results of our patients. This result highlighted the potential differences in clinical presentation between stop gain, frame shift, or splicing variants and missense variants.

## 4 Discussion

Laminin is an extracellular matrix protein and a major component of the basement membrane (8). During embryonic development, it is believed to mediate cell attachment, migration, and tissue organization by interacting with other extracellular matrix components. Structurally, laminin consists of three subunits (*α*, *β*, and *γ*) linked to one another via disulfide bonds. The *LAMA2* gene (OMIM: 156225) encodes the laminin α2 chain, a subunit of both laminin-2 (merosin) and laminin-4 (s-merosin). As a tissue-specific component of the extracellular matrix, the laminin α2 chain plays a critical role in myotube stability and the regulation of apoptosis (9). The *LAMA2* gene is located on chromosome 6q22.33 and contains 65 exons, which collectively encode a protein of 3,122 amino acids (10). According to data from the GTEx (Genotype-Tissue Expression) and HPA (Human Protein Atlas) databases, *LAMA2* is widely expressed across tissues (including skeletal muscle) during both embryonic development and adulthood. Variants in *LAMA2* lead to *LAMA2*-related disorders (*LAMA2*-RDs), which exhibit a broad clinical phenotypic spectrum, ranging from early-onset severe forms to late-onset mild progressive disease. These disorders include congenital muscular dystrophy with complete merosin deficiency (MDC1A), congenital muscular dystrophy with partial merosin deficiency, and autosomal recessive limb-girdle muscular dystrophy 23 (LGMDR23) (5). Its core clinical manifestations include early infantile motor developmental delay, marked elevation of serum creatine kinase (CK) levels, joint contractures, and progressive respiratory involvement. Consistent with these typical features, all patients in our study presented with motor developmental delay, elevated muscle enzyme levels, and ankle contractures (Table 1). According to the HPA and GTEx database, *LAMA2* also expressed in brain and cortex. The variants of loss of function (Lof) resulted the dysfunction of *LAMA2* protein and contributed to the white matter abnormalities which was also observed in our patients. This further highlighted the stop gain, frameshift, or splicing variants’ contribution to brain development.

In addition to the aforementioned clinical phenotypes, characteristic cranial imaging findings serve as an effective clinical screening tool for the *LAMA2*-related disease spectrum. Previous studies indicated that most patients present with periventricular white matter abnormalities, a finding potentially linked to the role of G proteins in peripheral nerve development and glial cell adhesion (11). Salvati et al. (12) reported that 93.6% of patients exhibited diffuse subcortical or periventricular white matter signal abnormalities, while approximately 36.2% had cortical developmental malformations.

Consistent with these prior observations, 80% of patients (4 out of 5) in our cohort displayed periventricular white matter abnormalities. Notably, one patient showed no such imaging changes. However, genetic testing for this patient identified compound heterozygous *LAMA2* variants: a maternal variant (c.5095del, p.Leu1699*) and a paternal variant (c.7147C>T, p.Arg2383*). Clinically, this patient presented with motor developmental delay, ankle contractures, and abnormal muscle enzyme levels. The absence of cerebral white matter abnormalities in this case may be attributed to the patient’s young age at the time of imaging; this hypothesis requires confirmation through long-term follow-up. Three patients experienced two or more seizures which characterized as motor seizures with loss of consciousness. Interictal electroencephalography demonstrated a slow background rhythm, with absence of epileptiform discharges. However, the prevalence of seizures in *LAMA2*-related disorders remains controversial in the literature: previous studies have suggested that intellectual disability and/or seizures are rare (13), while Camelo, Salvati, and colleagues have reported marked clinical phenotypic heterogeneity, with seizure rates ranging from 19.2% to 74%—and even proposed that seizures may represent a core symptom of *LAMA2*-related diseases (9, 11). The reasons for the differences in epilepsy incidence between our study and other studies may duo to multiple reasons including the differences in patient age distribution, follow-up duration, or epilepsy diagnostic criteria, etc.

Seizure types in these disorders are diverse, with generalized tonic-clonic seizures being the most common (12). A small number of cases present with focal motor seizures (with or without impaired consciousness), while typical or atypical absence seizures are rare, and myoclonic seizures are extremely uncommon. Notably, Guo et al. (14) did not document a history of epilepsy in 43 patients (from both domestic and international cohorts) they reviewed, this discrepancy may stem from incomplete case data or insufficient long-term follow-up. At our center, the three patients with seizures developed symptoms 3–8 years after their initial presentation. The ages at seizure onset were 91 months, 117 months, and 151 months (mean: 119.37 ± 36.43 months). Whereas, the mean onset age was 8.13 ± 5.36 years reported by Salvati et al. (12). These results support the temporal evolution of *LAMA2*-related disorders, emphasizing the need for dynamic clinical monitoring, as well as dietary and lifestyle guidance, throughout the disease course.

The specific mechanism of seizures in *LAMA2*-related disorders remains unclear. Camelo et al. (15) hypothesized that *LAMA2*-RD patients with cortical malformations, epilepsy, and intellectual disability harbor genetic variants affecting the laminin G (LG) domain of the laminin-α2 protein. In contrast, Salvati et al. (12) found no significant correlation between cortical developmental malformations and the age of seizure onset. Geranmayeh et al. (16) reported that complete or partial loss of laminin-α2 (merosin) correlates with clinical phenotype severity, age of disease onset, and rate of progression. Oliveira et al. (6) further noted that late-onset LAMA2-MD, most commonly associated with missense variants or in-frame deletions, often presents with brain imaging abnormalities, cognitive impairment, and refractory seizures. However, other studies have failed to identify a clear association between cognitive impairment, seizures, and brain structural abnormalities.

In recent years, research investigating genotype-phenotype correlations in *LAMA2*-related disorders has expanded. As of May 27, 2024, analysis of *LAMA2* genetic variants (summarized in Figure 5) revealed that single nucleotide variants (SNVs) accounted for approximately 83.8% of all variants, while deletions constituted 8.25%. Oliveira et al. (6) reported that stop-gain variants (premature termination codons, PTCs) are the most prevalent genotype in these disorders. Such variants are associated with the complete absence of laminin-α2 protein in muscle biopsies and correlate with the congenital muscular dystrophy type 1A (MDC1A) phenotype—the severe, early-onset form of *LAMA2*-related disease. In contrast, missense variants are less frequent; they typically result in partial laminin-α2 defects and are linked to milder phenotypic presentations. A single-center study conducted by the Department of Pediatrics at Peking University First Hospital analyzed 134 children with hereditary myopathies, among whom 22 were diagnosed with MDC1A. Genetic testing of these 22 MDC1A patients identified 8 cases with compound heterozygous variants [combining *LAMA2* SNVs and copy number variations (CNVs)] and 14 cases with compound heterozygous SNVs alone. Consistent with these findings, all patients in our cohort harbored compound heterozygous *LAMA2* variants (detailed in Table 1). Detailed variant site information was available for only two patients, one of whom carried a nonsense point mutation. As illustrated in Figure 3, the variants were localized to the laminin G-like domain, helix domain I, and laminin EGF-like domain regions of the laminin-α2 protein, respectively.

Notably, Oliveira et al.’s (6) work further corroborates genotype–phenotype associations in *LAMA2*-related disorders: stop-gain (PTC) variants are strongly associated with the MDC1A phenotype and complete merosin deficiency, whereas missense variants typically lead to partial merosin defects and milder disease manifestations. The truncating variants typically caused severe damaging effects of gene function, while missense potentially associated with mild damaging effects. The difference of functional effects among genotype may explain the phenotypic variation.

Similar genotype–phenotype associations were also observed in other genes, such as APC2 (17), CCDC22 (18), DLG3 (19), TANC2 (20), SRCAP (21), SZT2 (22).

To date, clinical studies on *LAMA2*-related disorders have primarily documented neuromuscular manifestations, which are limited to four well-characterized phenotypes (6, 23): (1) congenital muscular dystrophy type 1A (MDC1A) with complete merosin deficiency; (2) congenital muscular dystrophy with partial merosin deficiency; (3) late-onset autosomal recessive limb-girdle muscular dystrophy type 23 (LGMDR23); and (4) peripheral neuropathy. Notably, no clinical manifestations involving the ear, pituitary gland, adrenal gland, or uterus have been reported in the literature. These observations suggest that the clinical phenotypes associated with *LAMA2* variants may be broader than previously recognized. The spatiotemporal (tissue- and developmental stage-specific) expression patterns of *LAMA2* could help retroactively supplement our understanding of unrecognized *LAMA2*-related phenotypes. More importantly, these expression patterns may serve as a theoretical and prospective basis for expanding the clinical phenotype spectrum of *LAMA2*-related disorders, highlighting the need for further research into non-neuromuscular manifestations.

### 4.1 Limitation of this study

Firstly, this study only included 5 patients, which may lead to insufficient statistical power and limited representativeness, influencing to support the clinical heterogeneity of *LAMA2*-MD (*LAMA2*-related muscular dystrophy) and the rules of genotype–phenotype correlation. Secondly, since it was a retrospective study, muscle tissue samples from patients were not obtained to verify at the protein level which may affect the support of genotype–phenotype correlation analysis. Thirdly, the age range of the patients was relatively wide (age at first diagnosis: 3 months to 78 months), and there may be significant differences in the follow-up duration among different patients. Such imbalances in age and follow-up duration may interfere with the analysis results of “disease progression over time.” Finally, one patient carried a missense variant of VUS (c.6624G>C, p.Trp2208Cys). The speculation that this variant may affect protein function was only based on structural simulation and lacks experimental verification.

## 5 Conclusion

*LAMA2*-related disorders exhibit marked clinical heterogeneity and a progressive disease course over time. The temporal evolution of *LAMA2*-related disorders and the heterogeneous involvement of tissues/organs can be characterized by reviewing real-world clinical data records (CDRs) from modern hospital information systems. Additionally, the clinical phenotypic spectrum of these disorders can be retroactively refined using the spatiotemporal expression patterns of the *LAMA2* gene.

## Data Availability

The datasets presented in this article are not readily available due to ethical restrictions and privacy concerns related to human genetic/clinical data. Requests to access the datasets (raw clinical and processed data) should be directed to the corresponding author Xiongying Yu (yuxyjxeth@163.com).
